# A Generational Comparison of Physical and Laboratory Findings in Patients with Polyendocrine Metabolic Ovarian Syndrome in Japan

**DOI:** 10.3390/jcm15176710

**Published:** 2026-08-29

**Authors:** Saki Minato, Yuri Yamamoto, Hiroki Noguchi, Moeka Arata, Kou Tamura, Hidenori Aoki, Asuka Takeda, Ayana Takahashi, Tugsjargal Purevdorj, Ayaka Shinohara, Hiroaki Inui, Riyo Kinouchi, Kanako Yoshida, Toshiya Matsuzaki, Takeshi Iwasa

**Affiliations:** 1 Department of Obstetrics and Gynecology, Graduate School of Biomedical Sciences, Tokushima University, Tokushima 770-8053, Japan; shootingstar.53.mfn@gmail.com (S.M.);; 2 Department of Obstetrics and Gynecology, Yoshinogawa Medical Center, Tokushima 776-8511, Japan

**Keywords:** polyendocrine metabolic ovarian syndrome, polycystic ovary syndrome, metabolic syndrome, generational comparison, Japanese

## Abstract

**Background/Objectives**: Women with polyendocrine metabolic ovarian syndrome (PMOS) are known to be at higher risk of developing metabolic-related disorders. Here, we conducted a retrospective observational study to compare physical and laboratory findings associated with PMOS across different generations. **Methods**: We analyzed 470 medical records from 155 patients with PMOS from December 2002 to March 2023. Demographic, metabolic, and hormonal data were extracted and compared among age groups: <30, 30s, and ≥40 years. **Results**: The 30s group had significantly higher total cholesterol and glycated hemoglobin levels than the <30 group, while the ≥40 group had significantly higher diastolic blood pressure than both the <30 and 30s groups. The ≥40 group had significantly higher follicle-stimulating hormone and lower testosterone levels than both the <30 and 30s groups. Dehydroepiandrosterone sulfate levels and ovarian volume also differed significantly among the three groups, with both parameters decreasing with age. No significant differences in fasting glucose, fasting insulin, or homeostatic model assessment for insulin resistance were observed among the three groups. In multivariable linear mixed-effects models including age, BMI, and PMOS diagnostic criteria, BMI was independently associated with a broader range of metabolic parameters than age, whereas age was independently associated with follicle-stimulating hormone, testosterone, dehydroepiandrosterone sulfate, and ovarian volume. **Conclusions**: The present findings suggest that metabolic risk factors may emerge from a relatively young age in women with PMOS, with BMI showing broader associations with metabolic parameters than age. These observations support current recommendations for long-term metabolic monitoring and lifestyle management in women with PMOS.

## 1. Introduction

Polyendocrine metabolic ovarian syndrome (PMOS) was previously known as polycystic ovary syndrome (PCOS); however, the terminology was revised to PMOS in May 2026 [1]. The change reflects concerns that the term PCOS may be misleading, as it may imply the presence of pathological ovarian cysts and inadequately convey the diverse endocrine and metabolic features of the syndrome. This terminology may contribute to delayed diagnosis and fragmented care. PMOS is the most common reproductive endocrinopathies and is characterized by symptoms such as menstrual abnormalities, infertility, hyperandrogenism, hirsutism, insulin resistance, hyperinsulinemia, and obesity [2,3]. These symptoms are linked to the increased risk of various metabolic disorders, such as metabolic syndrome, diabetes, hypertension, and cardiovascular disease and infertility [4,5]. In particular, central obesity, glucose intolerance, dyslipidemia, and hypertension, the main parameters of metabolic syndrome, are known to increase the risk of developing metabolic diseases [6], and their prevalence tends to increase with age and the resultant accumulation of fat [7].

Several studies have shown that the clinical symptoms and physical findings of PMOS change with age, i.e., reproductive symptoms improve whereas metabolic characteristics worsen [8]. For example, the percentage of patients with regular menstrual cycles with PMOS has been reported to increase from 40.6% in women aged 30–35 years to 100% in those aged 51–55 years [8]. Similarly, serum levels of androgen, testosterone (T), androstenedione, and dehydroepiandrosterone sulfate (DHEAS) decrease with aging, whereas polycystic ovarian morphology, ovarian volume, and the number of antral follicles improve [9,10,11]. In addition, age-related changes in metabolic abnormalities have been reported in women with PMOS. For example, the risks for impaired glucose tolerance, hypertension, and type 2 diabetes increase with increasing age and body mass index (BMI) [12]. Similarly, regarding lipid metabolism, triglycerides (TG) and low-density lipoprotein cholesterol (LDL-C) have been shown to increase with age, while high-density lipoprotein cholesterol (HDL-C) has been shown to decrease [13]. However, inconsistent outcomes have been seen across studies [14]. Regardless, physicians should pay attention to the higher risk of various metabolic disorders in women with PMOS throughout their lifetime.

Although metabolic and reproductive characteristics and clinical symptoms, including the risk factors of related disorders, are known to change with age in women with PMOS, to our knowledge, few reports have comprehensively examined this issue from a variety of perspectives, especially in Japanese women with PMOS. Collecting such data is therefore considered important for the long-term management of PMOS. Given this background, its objective is to retrospectively compare physical and laboratory findings regarding metabolic and reproductive disorders in patients with PMOS across different generations.

## 2. Materials and Methods

### 2.1. Participants

In this study, 470 medical records from 155 patients with PMOS undergoing treatment or follow-up at Tokushima University Hospital in Japan from December 2002 to March 2023 (data from patients with multiple visits to the hospital were included) were analyzed. Concretely, 222 medical records from patients aged <30 years (<30 group), 202 from those in their 30s (30s group), and 46 from those aged ≥40 years (≥40 group) were used. The proportions of cases with repeated measurements in each age group were 162 of 222 cases (73.0%) in the <30 group, 152 of 202 cases (75.2%) in the 30s group, and 42 of 46 cases (91.3%) in the ≥40 group.

The diagnosis of PMOS was determined using the Japanese diagnostic criteria established by the Japan Society of Obstetrics and Gynecology (JSOG) in 1993 or 2007. In the 1993 criteria, all of the following criteria must be met for a diagnosis of PMOS: (1) irregular menstrual cycle/chronic anovulation, (2) high basal luteinizing hormone (LH) and normal basal follicle stimulating hormone (FSH), and (3) polycystic ovarian morphology. In the 2007 criteria, all of the following criteria must be met for a diagnosis of PMOS: (1) irregular menstrual cycle/chronic anovulation, (2) polycystic ovarian morphology (i.e., the presence of many small follicles detected by transvaginal ultrasonography, with at least one ovary containing 10 small follicles measuring 2–9 mm in diameter), and (3) hyperandrogenemia or high basal LH and normal basal FSH [15,16,17]. All patients with PMOS diagnosed according to the JSOG 1993 and 2007 criteria fulfilled the Rotterdam PMOS diagnostic criteria, which are widely used around the world. Conditions that present with symptoms similar to those of PMOS, including Cushing’s syndrome, adrenal enzyme abnormalities, and hyperprolactinemia, were excluded. Patients who had already been administered drugs to treat hypertension, diabetes, and dyslipidemia were excluded. Cases in which patients had received fertility medications or hormonal therapy within the preceding month were excluded.

### 2.2. Study Design

Physical measurements, lipid and glucose metabolism parameters, gonadotropin levels, and androgen-related parameters were extracted from the medical records of patients with PMOS. This retrospective observational study compared these parameters across three age groups: <30 years, in their 30s, and ≥40 years. This study was approved by the Ethics Review Committee of Tokushima University Hospital (approval No.: 4440, date of approval: 23 October 2023).

### 2.3. Physical Measurements and Clinical and Ultrasound Evaluation

Anthropometric measurements included height, weight, body fat percentage, waist and hip circumference, and BMI, which were taken on an outpatient basis. Height was measured using a manual height measuring device, while weight and body fat percentage were measured using an electronic body composition scale (InnerScan BC-210, TANITA, Tokyo, Japan). The accuracy of body fat percentage is within ±5%. In a relaxed standing position, waist circumference was measured horizontally at the level of the navel, and hip circumference was measured horizontally at the highest point of the buttocks. BMI was calculated as the weight in kilograms divided by the height in meters squared. Both systolic blood pressure (SBP) and diastolic blood pressure (DBP) were measured using an electronic sphygmomanometer while the patient was in a sitting position after resting for at least 5 min.

The presence of hirsutism or acne was determined based on patient complaints and the examining physician. The chief complaint at presentation and disease duration were extracted. Ovarian volume was calculated using transvaginal ultrasound (SONOVISTA, KONICA MINOLTA, Tokyo, Japan; frequency, 5–10 MHz) based on three-dimensional measurements of the ovaries and the standard simplified formula for an ellipsoid (0.5 × length × width × thickness). The mean volume of the left and right ovaries was then calculated. Ultrasound examinations were performed by obstetricians and gynecologists with at least two years of experience. Measurements were obtained either during menstruation or during the follicular phase in the absence of developing follicles > 10 mm in diameter.

### 2.4. Laboratory Measurements

Blood samples were taken after fasting for at least 10 h, at a time when no fertility drugs or hormones had been administered within a month, and after a transvaginal ultrasound scan confirmed no follicular development >10 mm. Serum levels of total cholesterol (TC), LDL-C, HDL-C, and TG—lipid profile parameters—and fasting blood glucose (FBG), fasting insulin (FINS), and glycated hemoglobin (HbA1c)—glucose metabolism parameters—were measured. In addition, homeostatic model assessment for insulin resistance (HOMA-IR), which is one of the parameters of insulin resistance, was calculated using the following formula: FBG (mg/dL) × FINS (µU/mL)/405.

Serum levels of LH, FSH, estradiol (E2), T, free testosterone (free T), and DHEAS were also measured, and the ratio of LH to FSH was calculated as LH divided by FSH and expressed as the LH/FSH ratio. Serum E2, LH and FSH levels were determined by cobas6000 (Roche Diagnostics K.K., Tokyo, Japan) based on the principle of electrochemiluminescence immunoassay (ECLIA). Serum T levels were determined by AIA-2000 (Tosoh Co., Tokyo, Japan) based on the principle of enzyme immunoassay (EIA). Serum free T levels were determined by AccuFLEXγARC-8010 (Immunotech S.R.O., Prague, Czech Republic) based on the principle of radioimmunoassay solid phase method. Serum DHEAS levels were determined by UniCel DxI 800 (Beckman Coulter inc., Tokyo, Japan) based on the principle of chemiluminescent enzyme immunoassay (CLEIA).

### 2.5. Statistical Analysis

Physical and laboratory parameters, except for age, were presented as medians and interquartile ranges, and the presence of hirsutism or acne as counts (%). Age was expressed as the mean and standard deviation. Intergroup comparisons were performed using linear mixed-effects models to account for within-subject correlations due to repeated measurements, with adjustment for multiple comparisons. The presence or absence of hirsutism and acne was compared using logistic generalized estimating equations (GEE). Associations between age, BMI, and diagnostic criteria and various metabolic and reproductive parameters were evaluated using linear mixed-effects models. Cases diagnosed according to the 1993 JSOG criteria were coded as 0, whereas those diagnosed according to the 2007 JSOG criteria were coded as 1. A two-sided *p*-value of <0.05 was considered statistically significant. All statistical analyses were performed using R version 4.6.1 (R Foundation for Statistical Computing, Vienna, Austria). Linear mixed-effects models were fitted using the lme4 package (version 2.0.6), with p-values calculated using the lmerTest package (version 3.2.1). Estimated marginal means and Tukey-adjusted pairwise comparisons were obtained using the emmeans package (version 2.0.4). Generalized estimating equation analyses were performed using the geepack package (version 1.3.13).

## 3. Results

The proportions of patients with menstrual irregularities or infertility as their chief complaint at presentation were 77.5% and 22.5% in the <30 group, 64.4% and 35.6% in the 30s group, and 95.7% and 4.3% in the ≥40 group, respectively. The median disease duration in the <30, 30s, and ≥40 groups was 25 (4–48) months, 54 (20–95) months, and 163 (115.75–225) months, respectively. The proportions of patients diagnosed according to the JSOG 1993 or the JSOG 2007 criteria were 22.5% and 77.5% in the <30 group, 14.9% and 85.1% in the 30s group, and 2.2% and 97.8% in the ≥40 group, respectively.

Table 1 presents the general characteristics and physical measurements of the <30, 30s, and ≥40 groups, together with the results of between-group comparisons, using linear mixed-effects models. The mean ages of the three groups were 24.9 ± 3.3, 33.5 ± 2.8, and 43.2 ± 3.4 years, respectively. Compared with the <30 group, the 30s group had a significantly greater waist circumference (*p* < 0.05). The ≥40 group had a significantly higher DBP than both the <30 group (*p* < 0.05) and the 30s group (*p* < 0.01). No significant differences were observed in height, weight, BMI, body fat percentage, hip circumference, or SBP.

Table 2 presents the lipid and glucose metabolic parameters of the <30, 30s, and ≥40 groups, together with the results of between-group comparisons using linear mixed-effects models. Compared with both the 30s group and the ≥40 group, the <30 group had significantly lower TC levels (*p* < 0.05 for both comparisons). Regarding HbA1c, the 30s group had significantly higher levels than the < 30 group, whereas the ≥ 40 group had significantly higher levels than both the <30 and 30s groups (*p* < 0.01 for all comparisons). No significant between-group differences were observed in LDL-C, HDL-C, TG, FBG, FINS, or HOMA-IR.

Based on physical measurements and lipid and glucose metabolism parameters, the percentages of patients who met the Adult Treatment Panel III (ATP III) diagnostic criteria for metabolic syndrome were 4.5% in the <30 group, 16.3% in the 30s group, and 17.4% in the ≥40 group.

Table 3 presents the reproductive parameters related to gonadotropins and androgens in the <30, 30s, and ≥40 groups, together with the results of between-group comparisons using linear mixed-effects models. The ≥40 group had significantly higher FSH and significantly lower T levels than both the <30 and 30s groups (*p* < 0.01 for all comparisons). Significant differences in DHEAS levels were observed between the groups, with significant differences between the <30 and 30s groups (*p* < 0.01), between the <30 and ≥40 groups (*p* < 0.01), and between the 30s and ≥40 groups (*p* < 0.05). Ovarian volume also differed significantly between the groups, with significant differences between the <30 and 30s groups (*p* < 0.05), between the <30 and ≥40 groups (*p* < 0.01), and between the 30s and ≥40 groups (*p* < 0.01). No significant differences were observed in E2, LH, the LH/FSH ratio, or free T.

**Figure 1:**
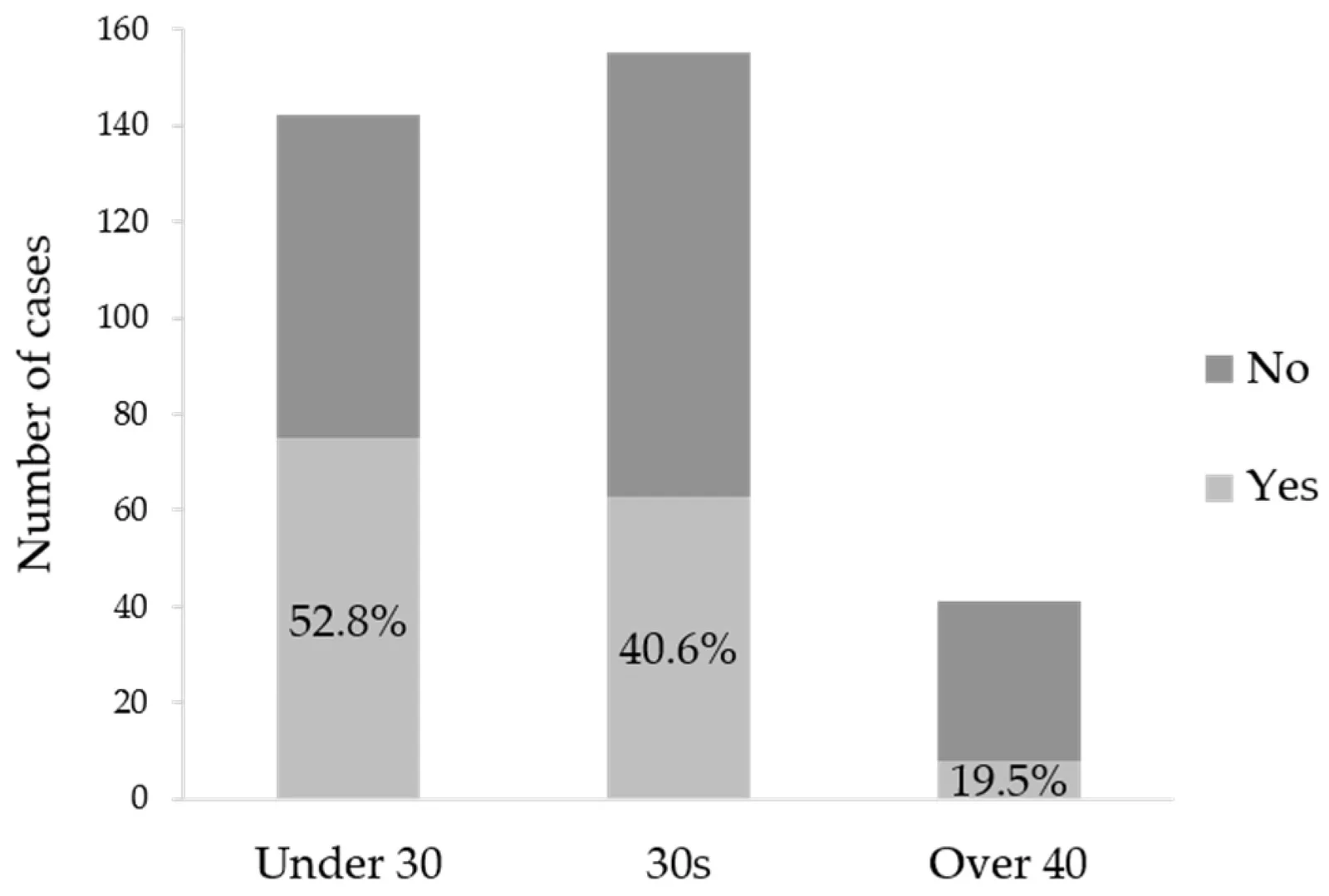
Presence of hirsutism or acne in the groups of under 30, 30s, and over 40. overall *p* value = 0.337.

The percentages of hirsutism or acne in the <30, 30s, and ≥40 groups are shown in Figure 1. Analysis using a logistic GEE accounting for repeated measurements revealed no significant differences among the three groups (overall *p* = 0.337).

Table 4 presents the results of linear mixed-effects models including age, BMI, and PMOS diagnostic criteria as covariates. After mutual adjustment for age, BMI, and diagnostic criteria, BMI was independently and positively associated with TC, LDL-C, TG, HbA1c, FBG, FINS, and HOMA-IR (*p* = 0.004 for TC; *p* < 0.001 for all other parameters) and independently and negatively associated with HDL-C (*p* < 0.001). Age was independently and positively associated with TC, LDL-C, and HbA1c (*p* < 0.001 for all) and independently and negatively associated with FINS (*p* < 0.001) and HOMA-IR (*p* = 0.026). Diagnostic criteria were independently associated with HbA1c, with patients diagnosed according to the 2007 JSOG criteria having significantly higher HbA1c levels than those diagnosed according to the 1993 JSOG criteria (*p* < 0.001).

Table 5 presents the results of reproductive parameters analyzed using linear mixed-effects models including age, BMI, and PMOS diagnostic criteria as covariates. After mutual adjustment for age, BMI, and diagnostic criteria, BMI was independently and negatively associated with LH (*p* = 0.020) and independently and positively associated with T (*p* = 0.017) and free T (*p* < 0.001). Age was independently and positively associated with FSH (*p* < 0.001) and independently and negatively associated with T, DHEAS, and ovarian volume (*p* < 0.001 for all). Diagnostic criteria were independently associated with DHEAS, with patients diagnosed according to the 2007 JSOG criteria having significantly higher DHEAS levels than those diagnosed according to the 1993 JSOG criteria (*p* = 0.025). No significant associations were observed between age, BMI, or diagnostic criteria and the LH/FSH ratio.

## 4. Discussion

This study compared and analyzed the characteristics of physical findings and clinical laboratory values, including factors related to metabolic and reproductive disorders, in Japanese patients with PMOS across different generations. Regarding metabolism-related factors, the following parameters were used for the diagnosis of Japanese metabolic syndrome: waist circumference, TG, HDL-C, SBP, DBP, and FBG [18].

Among these parameters, waist circumference was significantly lower in the <30 group compared to the 30s group but did not exceed the diagnostic criteria for metabolic syndrome (≥90 cm) in any group. Although no significant differences in weight, BMI, or body fat percentage were observed among the three groups, it is noteworthy that the mean BMI in the 30s group exceeded 25 kg/m^2^. The JSOG 1993 criteria do not include biochemical hyperandrogenemia, meaning that all women diagnosed using the JSOG 1993 criteria would have a high LH/FSH ratio. Because obese women with PMOS tend to have a lower LH/FSH ratio than lean women with PMOS, women diagnosed with PMOS using the JSOG 1993 criteria may be less obese than women meeting the JSOG 2007 criteria. In the present study, a higher proportion of women diagnosed according to the 1993 JSOG criteria were younger, which may have contributed to the lower BMI observed in the <30 group, although the difference was not statistically significant compared with the other two groups. Japanese women are often thin, and it has been reported that the proportion of Japanese women aged ≥18 years who have a BMI ≥ 25 kg/m^2^ tends to be smaller than that of other countries [19]. According to the 2023 National Health and Nutrition Survey in Japan [20], the proportion of Japanese women with a BMI ≥ 25 kg/m^2^ is only 11.8% among those aged 20–29 years, 12.4% among those aged 30–39 years, and 19.0% among those aged 40–49 years. Reflecting this trend, the prevalence of obesity in Japanese women with PMOS is lower than that in other countries. A recent nationwide Japanese survey reported that the percentage of women with PMOS with a BMI ≥ 25 kg/m^2^ was 26.0% [21], whereas previous reports indicated that the percentage of patients with PMOS with a BMI ≥ 25 kg/m^2^ was approximately 40–80% in other Asian countries [22,23,24,25] and 60–90% in people of European descent [26,27,28,29]. However, some studies have shown that the risk of metabolic disorders is high regardless of BMI and that obesity can worsen such conditions in Asian patients with PMOS. Therefore, physicians should be aware that the risk of metabolic disorders increases with age in Japanese women with PMOS.

Regarding blood pressure, all median values were within the normal ranges, but DBP was significantly higher in the ≥40 group than in the <30 and 30s groups. According to the 2023 National Health and Nutrition Survey in Japan [20], the proportion of Japanese women with normal blood pressure (SBP < 130 mmHg and DBP < 80 mmHg) who were not taking antihypertensive medication was 92.6% among those aged 20–29 years, 87.3% among those aged 30–39 years, and 80.4% among those aged 40–49 years, and blood pressure tended to increase with age. Although the causal relationship with aging cannot be established in this study, higher blood pressure observed in the ≥40 group compared with the other two groups may suggest that women with PMOS show signs of being prone to developing hypertension from a young age.

Regarding glucose and lipid metabolism, Liang et al. [30] reported positive correlations between age and both FBG and TG in women with PMOS aged 20–40 years. De Medeiros et al. [31] reported that FBG, TG, and TC increased with age in women with PMOS aged <40 years, and that FBS and TG were significantly higher in women with PMOS aged >25 years compared with controls. Previous reports have shown that women with PCOS exhibit impaired glucose tolerance after the age of 30 years [30,32,33]. In the present study, TC and HbA1c levels were significantly higher in the 30s group than in the < 30 group, whereas no significant differences in LDL-C, HDL-C, TG, or FBG were observed among the three groups. Regarding HDL-C, de Medeiros et al. [31] reported no significant age-related changes in women with PMOS aged <40 years, which was consistent with the findings of this study. However, there have been some contradicting reports. For example, Falcetta et al. [34] found that HDL-C levels were lower in women with PMOS aged <20 years than in those aged 21–30 years, and other studies with longer follow-up periods (>20 years) until postmenopausal age have reported age-related decreases in HDL-C [13,35]. Furthermore, in the present analysis using linear mixed-effects models, only TC, LDL-C, and HbA1c showed independent associations with age, whereas BMI was independently associated with all metabolic parameters examined. These findings suggest that BMI may be more broadly associated with glucose and lipid metabolism than age. Although the reasons underlying these discrepancies in outcomes between the present and previous studies are unclear, racial, ethnic, or geographical differences, and statistical issues might be involved.

In the present study, no significant differences in FINS or HOMA-IR were observed among the three groups. Generally, insulin resistance increases with age, and many similar trends have been confirmed in women with PMOS in previous studies. For example, de Medeiros et al. [31] reported that FINS and HOMA-IR increased with age in women with PMOS, even those aged <40 years, and Falcetta et al. [34] reported that the quantitative insulin sensitivity check index, a measure of insulin resistance, was lower in the >30 age group than in the 21–30 age group. However, some studies have also reported contradictory findings, e.g., that FINS or insulin resistance did not change over time in women with PMOS who maintained a stable BMI [33,36,37]. Furthermore, other studies have shown that FINS and HOMA scores decrease over time in women with PMOS [10,38]. The present linear mixed-effects model analysis revealed that both age and BMI were independently associated with FINS and HOMA-IR, with the absolute value of the standardized partial regression coefficient being higher for BMI than for age, suggesting a stronger association between BMI and HOMA-IR.

Numerous reports have shown that T and DHEAS levels decrease over time in women with PMOS during reproductive age [31,32,34,36,37], but do not change after menopause [39]. Declines in androgen levels with aging have been reported in not only women with PMOS, but also healthy controls [31], and despite various controversies, most data support the fact that androgen production declines with age, but not as much as in women without PMOS. It has recently been proposed that hyperandrogenism is associated with hypertension and metabolic abnormalities in both women with and without PMOS. Sutton-Tyrrell et al. [40] reported that among premenopausal and early perimenopausal women, the elevated free androgen index (FAI) was strongly associated with increased cardiovascular risk factors (i.e., higher insulin, glucose, and hemostatic and inflammatory markers and adverse lipids), even after adjusting for BMI. Similarly, Chen et al. [41] reported that T and FAI are significantly positively correlated with blood pressure, independent of insulin resistance, obesity, and dyslipidemia. In addition, a meta-analysis found that the incidence of metabolic syndrome is significantly higher in women with PMOS than in those without hyperandrogenism [42]. These previous findings suggest that women with PMOS show a decreasing trend in androgen production with age, but not to the same extent as controls, and therefore, maintain a hyperandrogenic condition from younger age, resulting in an increased risk of metabolic disorders.

Previous reports have indicated that the incidence of hirsutism or acne decreases with age [30]. Conversely, de Medeiros et al. [31] reported that acne, but not hirsutism, tended to decrease with age in women with PMOS, and van Keizerswaard et al. [8] reported that neither the incidence of acne and/or hirsutism nor the modified Ferriman–Gallwey score changed with age in women with PMOS. In this study, no significant differences in the incidence of hirsutism or acne were observed among the three groups, consistent with the findings of van Keizerswaard. Interestingly, it has been reported that although androgen levels in women with PMOS tend to decrease over time, hyperandrogenic hirsutism may be persistent even after menopause [43], and that hirsutism is not correlated with serum T and DHEAS levels [44]. The incidence of hirsutism in Japanese women with PMOS is lower than that in other countries, with a recent nationwide Japanese survey reporting a 13.5% incidence of hirsutism among patients with PMOS [21]. It is possible that changing trends in hyperandrogenic phenotypes with age also differ among ethnic groups.

Many previous studies have reported that ovarian volume in women with PMOS decreases with age [8,37]. In the present study, ovarian volume decreased with age, similar to the findings of many previous studies [8,37]. Although an age-related decrease in ovarian volume has also been observed in women without PMOS [45,46], Ahmad et al. [47] reported that women with PMOS had a slower decline rate in ovarian volume compared with non-PMOS controls. In addition, one study reported that ovarian volume did not change over time in women with PMOS aged <39 years [31], and a 20-year follow-up study found that ovarian volume was significantly decreased at around 40 years of age in women with PMOS [37], suggesting that ovarian volume in women with PMOS may decrease with age. Consistent with this finding, ovarian volume decreased with increasing age across the three groups in the present study.

This study has several limitations. Because this study was a retrospective observational study based on data collected over a 20-year period rather than a longitudinal study, it cannot directly assess longitudinal age-related changes. Furthermore, the analysis included multiple observations from the same individuals, which differs from the design of conventional cross-sectional studies. The observed differences between age groups may reflect general aging, cohort, or period effects (e.g., changes in diagnostic criteria, clinical practice, or patient characteristics over time) rather than PCOS-specific processes, and therefore age-related differences should be interpreted with caution. Because an age-matched healthy control group was not included, it remains unclear whether the observed differences reflect changes specific to PMOS or general age-related physiological changes. Furthermore, the lack of objectivity in the diagnoses of hirsutism and acne, as well as the very small number of participants aged ≥40 years compared with the other two groups, may have influenced our results. In the analysis, multiple medical records of the same individual were included, limiting statistical independence. Selection bias cannot be excluded because the participants were women who were referred to and attended Tokushima University Hospital. Because we focused on Japanese patients with PMOS, we were not able to evaluate the potential effects of racial and ethnic differences. The patients with PMOS in this study were diagnosed based on the Japanese PMOS diagnostic criteria (JSOG 1993 or 2007). As mentioned above, PMOS diagnosed using the Japanese criteria always fulfill the Rotterdam diagnostic criteria; however, there may be a bias toward certain phenotypes and symptoms. Finally, because this was a long-term study spanning 20 years, it is inevitable that some of the diagnostic and measurement instruments changed during the study period.

## 5. Conclusions

In summary, several metabolic parameters were significantly higher in the older age groups among Japanese women with PMOS, although no significant differences were observed in some parameters, including TG and FBG. In the multivariable linear mixed-effects models, BMI was independently associated with a broader range of metabolic parameters than age, suggesting that BMI may be an important factor associated with metabolic abnormalities in women with PMOS. In contrast, reproductive parameters, including FSH, T, DHEAS, and ovarian volume, showed independent associations with age. The present findings suggest that women with PMOS may begin to accumulate metabolic risk factors from a relatively young age. These observations support current recommendations for long-term metabolic monitoring and lifestyle management in women with PMOS.

## Figures and Tables

**Table 1:** General characteristics among the <30, 30s, and ≥40 age groups and analysis results from linear mixed-effects models.

	Under 30 Group (n = 222)	30s Group (n = 202)	Over 40 Group (n = 46)	Overall *p* Value	*p* Value Under 30 vs. 30s	*p* Value Under 30 vs. Over 40	*p* Value 30s vs. Over 40
Age (years)	24.9 ± 3.3	33.5 ± 2.8	43.2 ± 3.4	―	―	―	―
Height (cm)	161 (154–165)	156.5 (153–160)	158 (154–158)	NS	NS	NS	NS
Weight (kg)	52.4 (47.8–64.7)	61.4 (49.28–72.68)	56.1 (47.3–66.88)	NS	NS	NS	NS
BMI (kg/m^2^)	20.8 (18.83–24.53)	25.05 (20.16–29.62)	22.41 (18.9–29.21)	NS	NS	NS	NS
Body fat percentage (%)	26.6 (22.48–35.23)	35.3 (25.28–42.65)	29.95 (26–39.8)	NS	NS	NS	NS
Waist (cm)	68.8 (64.55–78.8)	81 (70.4–92.2)	82 (70–87.25)	<0.05	<0.05	NS	NS
Hips (cm)	90.75 (85.95–97.13)	95.4 (88–102.5)	94 (87.05–98.28)	NS	NS	NS	NS
SBP (mmHg)	113 (104–123)	119 (106–130)	114 (104–122.75)	NS	NS	NS	NS
DBP (mmHg)	70.5 (64–76)	74 (65–81.5)	72 (64–80)	<0.05	NS	<0.05	<0.01

Note: Age is presented as mean ± SD. Other data are presented as median (inter-quartile range). BMI, Body mass index; SBP, systolic blood pressure; DBP, diastolic blood pressure; NS, not significant.

**Table 2:** Lipid and glucose metabolic parameters among the <30, 30s, and ≥40 age groups and analysis results from linear mixed-effects models.

	Under 30 Group (n = 222)	30s Group (n = 202)	Over 40 Group (n = 46)	Overall *p* Value	*p* Value Under 30 vs. 30s	*p* Value Under 30 vs. Over 40	*p* Value 30s vs. Over 40
TC (mg/dL)	194.5 (175–214)	200 (184–220.75)	212 (198–228)	<0.05	<0.05	<0.05	NS
LDL-C (mg/dL)	110 (94–128.75)	122 (97–137.5)	132.5 (109.25–139)	NS	NS	NS	NS
HDL-C (mg/dL)	73 (58–89)	68 (52–83.25)	78 (57.25–89)	NS	NS	NS	NS
TG (mg/dL)	75 (54.25–100)	82.5 (57–133.75)	84 (60.25–154.75)	NS	NS	NS	NS
HbA1c (%)	5.2 (5.0–5.4)	5.3 (5.1–5.5)	5.3 (5.18–5.5)	<0.01	<0.01	<0.01	<0.01
FBG (mg/dL)	92 (87–96)	93 (89–100)	91 (84–96)	NS	NS	NS	NS
FINS (μU/mL)	6.1 (4–10.1)	6.3 (3.7–12.2)	4.5 (1.8–8.8)	NS	NS	NS	NS
HOMA-IR	1.43 (0.9–2.27)	1.43 (0.86–3.07)	0.91 (0.4–2.16)	NS	NS	NS	NS

Note: Data are presented as median (inter-quartile range). TC, total cholesterol; LDL-C, low-density lipoprotein cholesterol; HDL-C, high-density lipoprotein cholesterol; TG, triglycerides; HbA1c, hemoglobin A1c; FBG, fasting blood glucose; FINS, fasting insulin; HOMA-IR, homeostatic model assessment for insulin resistance; NS, not significant.

**Table 3:** Gonadotropins and androgen-related parameters among the <30, 30s, and ≥40 age groups and analysis results from linear mixed-effects models.

	Under 30 Group (n = 222)	30s Group (n = 202)	Over 40 Group (n = 46)	Overall *p* Value	*p* Value Under 30 vs. 30s	*p* Value Under 30 vs. Over 40	*p* Value 30s vs. Over 40
E2 (pg/mL)	49 (37–62)	49 (36–62)	39 (21.2–61)	NS	NS	NS	NS
LH (mIU/mL)	8.5 (5.88–12.13)	7.8 (4.9–11.5)	5.9 (3.85–10.68)	NS	NS	NS	NS
FSH (mIU/mL)	6.5 (5.48–7.8)	5.9 (4.9–7.2)	6.6 (5.9–9.4)	<0.01	NS	<0.01	<0.01
LH/FSH ratio	1.30 (0.85–1.86)	1.45 (0.83–1.95)	0.68 (0.43–1.15)	NS	NS	NS	NS
T (ng/mL)	0.4 (0.3–0.6)	0.4 (0.3–0.53)	0.3 (0.2–0.4)	<0.01	NS	<0.01	<0.01
free T (pg/mL)	1.1 (0.7–1.7)	1.0 (0.6–1.8)	0.9 (0.5–1.3)	NS	NS	NS	NS
DHEAS (ng/mL)	1980 (1450–2640)	1690 (1357.5–2150)	1280 (560–1890)	<0.01	< 0.01	<0.01	<0.05
Ovarian volume (cm^3^)	8.66 (6.72–12.05)	9.13 (6.75–11.58)	3.9 (3.06–5.81)	<0.01	< 0.05	<0.01	<0.01

Note: Data are presented as median (inter-quartile range). E2, estradiol; LH, luteinizing hormone; FSH, follicle-stimulating hormone; T, testosterone; free T, free testosterone; DHEAS, dehydroepiandrosterone sulfate; NS, not significant.

**Table 4:** Associations of age, BMI, and PMOS diagnostic criteria with lipid and glucose metabolic parameters in linear mixed-effects models.

Outcome	Predictor	β	95% CI	*p* Value
TC	Age	0.27	0.13 to 0.41	<0.001
	BMI	0.25	0.08 to 0.43	0.004
	Diagnostic criteria ^†^	0.006	−0.08 to 0.09	0.884
LDL-C	Age	0.26	0.14 to 0.38	<0.001
	BMI	0.40	0.24 to 0.56	<0.001
	Diagnostic criteria ^†^	−0.007	−0.07 to 0.06	0.834
TG	Age	0.03	−0.09 to 0.15	0.637
	BMI	0.54	0.39 to 0.68	<0.001
	Diagnostic criteria ^†^	0.02	−0.06 to 0.09	0.646
HbA1c	Age	0.39	0.28 to 0.50	<0.001
	BMI	0.29	0.14 to 0.44	<0.001
	Diagnostic criteria ^†^	0.11	0.06 to 0.17	<0.001
FBG	Age	0.09	−0.03 to 0.21	0.139
	BMI	0.34	0.19 to 0.49	<0.001
	Diagnostic criteria ^†^	0.05	−0.01 to 0.12	0.119
FINS	Age	−0.17	−0.27 to −0.08	<0.001
	BMI	0.82	0.72 to 0.93	<0.001
	Diagnostic criteria ^†^	0.02	−0.04 to 0.08	0.473
HOMA-IR	Age	−0.11	−0.21 to −0.01	0.026
	BMI	0.80	0.69 to 0.91	<0.001
	Diagnostic criteria ^†^	0.02	−0.04 to 0.08	0.508

Note: β, Standardized partial regression coefficient; CI, confidence interval; BMI, Body mass index; TC, total cholesterol; LDL-C, low-density lipoprotein cholesterol; TG, triglycerides; HbA1c, hemoglobin A1c; FBG, fasting blood glucose; FINS, fasting insulin; HOMA-IR, homeostatic model assessment for insulin resistance. ^†^: PMOS patients were diagnosed according to the JSOG 1993 criteria until April 2007 and according to the JSOG 2007 criteria from May 2007 onward. Cases diagnosed according to the JSOG 1993 criteria were assigned a value of 0, whereas those diagnosed according to the JSOG 2007 criteria were assigned a value of 1.

**Table 5:** Associations of age, BMI, and PMOS diagnostic criteria with reproductive parameters related to gonadotropins and androgens in linear mixed-effects models.

Outcome	Predictor	β	95% CI	*p* Value
LH	Age	0.04	−0.13 to 0.20	0.660
	BMI	−0.22	−0.40 to −0.03	0.020
	Diagnostic criteria ^†^	−0.05	−0.17 to 0.06	0.345
FSH	Age	0.27	0.12 to 0.42	<0.001
	BMI	−0.06	−0.22 to 0.09	0.411
	Diagnostic criteria ^†^	−0.10	−0.22 to 0.01	0.071
LH/FSH ratio	Age	−0.08	−0.24 to 0.08	0.323
	BMI	−0.13	−0.31 to 0.05	0.155
	Diagnostic criteria ^†^	0.02	−0.09 to 0.13	0.697
T	Age	−0.35	−0.50 to −0.20	<0.001
	BMI	0.20	0.04 to 0.36	0.017
	Diagnostic criteria ^†^	0.05	−0.03 to 0.14	0.232
free T	Age	0.06	−0.09 to 0.22	0.42
	BMI	0.32	0.15 to 0.49	<0.001
	Diagnostic criteria ^†^	−0.10	−0.20 to 0.00	0.06
DHEAS	Age	−0.33	−0.45 to −0.22	<0.001
	BMI	0.14	−0.02 to 0.29	0.078
	Diagnostic criteria ^†^	0.07	0.009 to 0.14	0.025
Ovarian volume	Age	−0.41	−0.56 to −0.27	<0.001
	BMI	0.004	−0.17 to 0.18	0.960
	Diagnostic criteria ^†^	−0.03	−0.11 to 0.06	0.553

Note: β, Standardized partial regression coefficient; CI, confidence interval; BMI, Body mass index; LH, luteinizing hormone; FSH, follicle-stimulating hormone; T, testosterone; free T, free testosterone; DHEAS, dehydroepiandrosterone sulfate. ^†^: PMOS patients were diagnosed according to the JSOG 1993 criteria until April 2007 and according to the JSOG 2007 criteria from May 2007 onward. Cases diagnosed according to the JSOG 1993 criteria were assigned a value of 0, whereas those diagnosed according to the JSOG 2007 criteria were assigned a value of 1.

## Data Availability

Data are available from the corresponding author upon reasonable request.

## Abbreviations

The following abbreviations are used in this manuscript:

PMOS	Polyendocrine Metabolic Ovarian Syndrome
PCOS	Polycystic Ovary Syndrome
T	Testosterone
DHEAS	Dehydroepiandrosterone Sulfate
BMI	Body Mass Index
TG	Triglycerides
LDL-C	Low-density Lipoprotein Cholesterol
HDL-C	High-density Lipoprotein Cholesterol
JSOG	The Japan Society of Obstetrics and Gynecology
LH	Luteinizing Hormone
FSH	Follicle Stimulating Hormone
SBP	Systolic Blood Pressure
DBP	Diastolic Blood Pressure
TC	Total Cholesterol
FBG	Fasting Blood Glucose
FINS	Fasting Insulin
HbA1c	Glycated Hemoglobin
HOMA-IR	Homeostatic Model Assessment for Insulin Resistance
E2	Estradiol
free T	Free Testosterone
ECLIA	Electrochemiluminescence Immunoassay
EIA	Enzyme Immunoassay
CLEIA	Chemiluminescent Enzyme Immunoassay
